# Oxidative Imbalance and Kidney Damage in Cafeteria Diet-Induced Rat Model of Metabolic Syndrome: Effect of Bergamot Polyphenolic Fraction

**DOI:** 10.3390/antiox8030066

**Published:** 2019-03-16

**Authors:** Daniele La Russa, Francesca Giordano, Alessandro Marrone, Maddalena Parafati, Elzbieta Janda, Daniela Pellegrino

**Affiliations:** 1 Department of Pharmacy, Health and Nutritional Sciences, University of Calabria, 87036 Rende, Italy; daniele.larussa@unical.it (D.L.R.); francesca.giordano@unical.it (F.G.); 2 LARSO (Analysis and Research on Oxidative Stress Laboratory), University of Calabria, 87036 Rende, Italy; alexbrown993@gmail.com; 3 Department of Biology, Ecology and Earth Sciences, University of Calabria, 87036 Rende, Italy; 4 Department of Health Sciences, Univ. “Magna Graecia” (Campus Germaneto), 88100 Catanzaro, Italy; mparafati@unicz.it (M.P.); janda@unicz.it (E.J.)

**Keywords:** oxidative stress, biological antioxidant potential, kidney damage, cafeteria diet, bergamot polyphenolic fraction

## Abstract

Obesity is a potent risk factor for kidney disease as it increases the possibility of developing diabetes and hypertension, and it has a direct impact on the development of chronic kidney disease and end-stage renal disease. In this study, we tested the effect of bergamot polyphenolic fraction in a cafeteria with diet-fed rats, an excellent experimental model for studying human metabolic syndrome, as it is able to induce severe obesity with insulin resistance and high plasma triglyceride levels more efficiently than a traditional lard-based high-fat diet used in rodent models. We analyzed the plasmatic oxidative balance by photometric tests, and the expression of cytoplasmic antioxidant enzymes (superoxide dismutase 1 and glutatione S-tranferasi P1) and apoptotic markers (Caspase 8 and 9) in kidney tissues by Western blot analysis. Our results clearly showed that the cafeteria diet induces a marked pro-oxidant effect: significant reduction of plasmatic antioxidant capacity; downregulation of cytoplasmic antioxidant enzymes expression; and activation of apoptotic pathways. All these hallmarks of redox disequilibrium were mitigated by treatment with polyphenolic fraction of bergamot, highlighting its antioxidant effect in the metabolic syndrome. Our data show that the link between obesity and renal damage could be represented by oxidative stress.

## 1. Introduction

The prevalence of overweight and obesity is constantly growing worldwide, even in low and middle income countries [1]. The most alarming aspect of this pandemic of obesity is that increasingly involves adolescents and children [2]. These findings highlight the necessity for development of effective food-policy actions focused on the key determinant of obesity, a healthy diet. Epidemiologic studies have highlighted the strong correlation between high body mass index (BMI) and several chronic diseases, including cardiovascular disease [3,4], cancers [5], and renal diseases [6,7,8]. Chronic kidney disease (CKD) represents a major public health concern and the main consequences include loss in renal function and cardiovascular complications [9]. 

Obesity is a potent risk factor for the development of new-onset kidney disease as it increases the possibility of developing diabetes and hypertension, and it has a direct impact on the development of CKD and end-stage renal disease (ESRD). The mechanisms whereby obesity may cause or exacerbate CKD remain unclear. It has been hypothesized that, to meet the high metabolic needs of the increase in body weight, a compensatory mechanism of hyperfiltration occurs with consequent increase in intraglomerular pressure that can damage the kidney structure and raise the risk of developing CKD in the long-term [6]. Indeed, observational studies showed that metabolically healthy obese subjects (without diabetes or hypertension) present a high risk of developing CKD, suggesting that obesity per se may cause renal dysfunction and kidney damage [10]. In particular, higher visceral adipose tissue has been associated with renal damage and great mortality in CKD patients, highlighting a direct role of visceral adiposity in organ damage [11,12,13]. Interestingly, also non-alcoholic fatty liver disease (NAFLD), a pathology characterized by fat accumulation in the liver in the absence of significant alcohol intake, was associated with an increased risk of CKD [14]. This harmful effect is probably due to the endocrine activity of adipose tissue via production of several adipokines involved in the development of inflammation, oxidative stress, abnormal lipid metabolism, increased production of insulin and insulin resistance [6]. Increased ROS production was observed in obesity and is closely linked to the development of metabolic syndrome and diabetes [15]. In addition, adiposity may be a potent, independent amplifier to the inflammatory and oxidative milieu already present in CKD [16].

Several studies have investigated the beneficial properties of various phytochemicals with particular attention to both antioxidant and anti-inflammatory activities. To date, existing studies appear encouraging, but results are premature to translate into clinical practice [17]. In particular, flavonoids, a family of polyphenols found abundantly in fruits, vegetables, nuts, whole-grains and vegetable oils consumed by humans, have been extensively studied and their beneficial effects have been documented in many diseases [18,19,20,21,22,23,24,25]. Citrus fruits and their juices show a high content of flavonoids and their nutraceutical efficacy has been shown in many studies. Bergamot (*Citrus bergamia Risso et Poiteau*) and bergamot-derived extracts, such as bergamot polyphenol fraction (BPF) have attracted a considerable attention due to its peculiar composition and the highest content of Citrus flavonoids, such as naringin, hesperidin and neoeriocitrin [26,27]. Its lipid-lowering, anti-diabetic, anti-inflammatory, and autophagy-stimulating activity have been confirmed in both animal models and clinical studies [28,29,30,31,32]. Although the metabolic effects of BPF suggest an underlying redox-balancing activity, this property has not been properly evaluated and documented so far. In addition, some authors have proposed a new mechanism of how food antioxidants exert their health-protective effects, i.e., the oxidative activation of the Nrf2 signaling pathway [33]. This mechanism, called “para-hormesis”, keeps antioxidant enzymes at the optimal levels consistent with good health [33]. In light of this, additional research is necessary to establish how bergamot-derived extracts interact with human physiological and pathological processes and what level of consumption (metabolism/excretion) is required to achieve health benefits.

In this work, we used a cafeteria (CAF) diet-fed rats, an excellent experimental model for studying human metabolic syndrome, as it is able to induce severe obesity with insulin resistance and high plasma triglyceride levels than a traditional lard-based high-fat diet used in rodent models [32]. To test the bergamot effect, we administered BPF to CAF-diet-fed rats. We hypothesized that the increased oxidative stress in obesity models may contribute to organ damage through activation of apoptosis signaling pathways and that BPF could have beneficial antioxidant effects. In order to test this assumption, we evaluated in our experimental models (i) plasmatic pro-oxidant/antioxidant status, (ii) expression of cytoplasmic antioxidant enzymes (SOD1 and GSTP1) and (iii) extrinsic and intrinsic apoptotic pathways.

## 2. Materials and Methods

### 2.1. Animals

Five-week-old male Rcc:Han WIST rats (*n* = 26; Harlan Laboratories, Indianapolis, IN, USA) were housed under controlled lighting (lights on at 7:00 a.m. and lights off at 7:00 p.m.) and temperature (20 ± 2 °C) conditions. The animals had access to water and were fed ad libitum with standard chow diet (SC, Harlan Teklad) for 3 weeks before assignment to one of four experimental groups. Ethics Statement: this animal study was approved by a local animal welfare committee and by the Italian Ministry of Health (project code: 01-24/09/2013), according to Legislative Decree 116/1992, which was in force when the study was proposed (before 4 March 2014). All surgery was performed under anesthesia and all efforts were made to minimize animal suffering.

### 2.2. Experimental Design

Rats (*n* = 26), at 8 weeks of age, were randomly assigned to two basic experimental groups: CAF diet group (*n* = 15) and control (CTR) diet group (*n* = 11). These two groups were subdivided into two subgroups: one received a BPF supplement (~50 mg/kg body weight/day) in drinking water (BPF, *n* = 6; BPF + CAF, *n* = 8) and the other received drinking water without BPF (CTR, *n* = 5; CAF, *n* = 7). The experimental diets administration started after a week and lasted 91–95 days until the day of sacrifice. Food consumption and body weight gain were monitored weekly for 13 weeks.

### 2.3. Diets and Supplement

The CAF diet comprised cookies (sweet or briny), milk chocolate, cereals, potato chips, processed meats, condensed milk with sugar, high-fat cheese (parmesan or provolone), and so on, were provided in excess. The CAF diet (75 kcal/rat/day) was offered in addition to standard chow (SC) diet ad libitum every 2–3 days. Each time a mix of snacks (salty and sweet) was supplied to stimulate hyperphagy. The snack consumption was monitored weekly by weighing them before and after consumption (corrected for drying), in order to calculate the amounts ingested of each one in all cages. According to the product labels’ information, CTR diet provided an energy value of 3.0 kcal/g, against the mean 4.2 ± 1.1 kcal/g of the items included in CAF diet [30]. BPF, as previously prepared and characterized for polyphenol content [26,30], was kindly provided by Herbal and Antioxidant Derivatives srl. (Polistena, RC, Italy). BPF contains 40% of flavonoids. The remaining part of BPF is a mixture of other polyphenols (mainly catechins, salts, fatty acids, carbohydrates and other compounds ([27] and unpublished observations). Neohesperidose-linked flavanones, such as naringin, neoeriocitrin and neohesperidin, account for over 60% of all flavonoids. BPF was provided (diluted in drinking water) daily or every 2 days in the BPF and BPF + CAF groups. We did not observe any change in pH, color and taste within 48 h. The consumption of water and BPF was monitored daily or every 2 days to calculate the daily intake of BPF. The BPF concentration in drinking water was progressively adjusted to the mean body mass in the cage to ensure a mean 50 mg/kg/rat/day dose over a 3-month period. This dose was five times higher than the previously tested dose in humans (1000 mg/100 Kg = 10 mg/Kg [28]) to account for five times higher metabolic rate in rodents according to Khan and coworkers [34].

### 2.4. Blood and Tissue Collection

At week 14, the animals were sacrificed under Zoletil (80 mg/kg) and Dormitor anesthesia for blood and tissue collection. The blood was collected by cardiac puncture in appropriate blood collection tubes, centrifuged (1700× *g*; 10 min at room temperature) and plasma were stored at −80 °C until use. The animals were perfused with 150 mM NaCl solution to remove excess of blood and to collect organs. 

### 2.5. Blood Analysis

In the serum, the following parameters were determined: triglycerides, total and low-density lipoprotein (LDL) cholesterol, glucose, creatinine, blood urea nitrogen (BUN), alanine aminotransferase, aspartate aminotransferase, gamma glutamyltransferase (GGT), total- (T-Bil) and direct bilirubin (bilirubin-D). The analyses were performed using commercial reagents on a Dimension EXL analyzer (Siemens Healthcare Diagnostics s.r.l., Milan, Italy). Routine blood counts were performed on EDTA-treated samples on Advia 2120 blood cell counter (Siemens, Erlangen, Germany).

### 2.6. Measurement of Plasma Oxidative Status

Plasma and tissue oxidative status determinations were measured by using photometric measurement kits and a free radical analyzer system with a spectrophotometric device reader (FREE Carpe Diem, Diacron International, Grosseto, Italy), which are routinely used in our laboratory [35,36,37,38]. Plasma oxidative stress was assayed using a Diacron-reactive oxygen metabolite (dROM) test. Results are expressed in Carratelli Units (UC; 1 UC = 0.8 mg/L of hydrogen peroxide). Total plasma antioxidant capacity was assayed using a biological antioxidant capacity (BAP) test. Results are expressed in µmol/L of the reduced ferric ions.

### 2.7. Western Blot and Densitometric Analysis

Tissue samples (800 mg) were prepared in a solution containing RIPA buffer (1.6 mL) and protease inhibitor cocktail (Sigma, St Louis, MO, USA), and then centrifuged at 14,000 rpm for 20 min at 4 °C. Protein quantification was performed with Bradford reagent kit (Sigma, St Louis, MO, USA). Proteins (50 µg) were heated (five minutes) in Laemmli buffer (Sigma, St Louis, MO, USA), separated by SDS-PAGE in a Bio-Rad Mini Protean III, and electroblotted onto nitrocellulose membrane (NitroBind, Maine Manufacturing, Sanford, ME, USA) using a mini trans-blot (Bio-Rad Laboratories, Hercules, CA, USA). Membrane was blocked in TBS-T buffer (5% non-fat dry milk). Blots were incubated with primary antibodies diluted in TBS-T overnight at 4°C (SOD1, polyclonal goat antibody; GSTP1, monoclonal mouse antibody; pJNK, monoclonal mouse antibody; Caspase 8, monoclonal mouse antibody; Caspase 9, polyclonal rabbit antibody); blots were incubated with peroxidase linked secondary antibodies for 1 h at room temperature. All antibodies were supplied by Santa Cruz Biotechnology, Inc., Dallas, USA. Immunodetection was obtained by an enhanced chemiluminescence kit (ECL Plus, GE Healthcare Amersham, Buckinghamshire, UK) and X-ray Films (Hyperfilm ECL, GE Healthcare Amersham, Buckinghamshire, UK). Digitalized immunoblots were subjected to densitometric analysis by WCIF Image J based on 256 grey values (0 ¼ white; 256 ¼ black) and results were expressed as means ± SE (standard error) of five determinations for each sample.

### 2.8. Statistical Analysis

Data was analyzed using the GraphPad/Prism version 5.01 statistical software (SAS Institute, Abacus Concept, Inc., Berkeley, CA, USA). Statistical differences were examined using two-way analysis of variance (ANOVA) followed by the Bonferroni multiple comparisons test. Data are expressed as the mean ± SE. 

## 3. Results

### 3.1. Effects of CAF Diet and BPF Treatment on Obesity and Blood Parameters

In laboratory rodents, obesity is defined as the achievement of a 20% increase in body mass index [39]. The CAF diet rapidly induces weight gain and obesity and after 14 weeks of treatment we found an increase in body weight of 31.97%. The BPF supplementation significantly reduced the final body weight in our obesity model indeed BPF + CAF group presents an increase in body weight of 19.46% with respect to CTR group. No significant effects of BPF on body weight were observed in animals fed SC diet and consuming daily bergamot polyphenols (BPF group) (Table 1). Concerning plasmatic biochemical profiles after 14 weeks of treatment, CAF diet led to a significant increase in blood glucose levels (+52.17%) with respect to CTR rats. Similarly, CAF diet significantly upregulated triglycerides level (+51.4%). Interestingly, our CAF diet protocol did not alter total and HDL cholesterol levels, while we observed a significant reduction of LDL-cholesterol in all treated groups. Triglyceridemia, significantly upregulated in CAF group, was potently reduced in BPF + CAF rats (−28.3%, *p* = 0.006), in agreement with our previous observations [28]. BPF also reduced blood glucose levels in CAF animals (−11.5%, *p* = 0.044). No significant differences in any relevant hematologic parameter were observed between the CTR and BPF groups (Table 1). The analysis of creatinine levels shows no variation in any of the experimental groups, indicating that renal function is preserved (Table 1).

### 3.2. Plasmatic Oxidative Status

**Figure 1:**
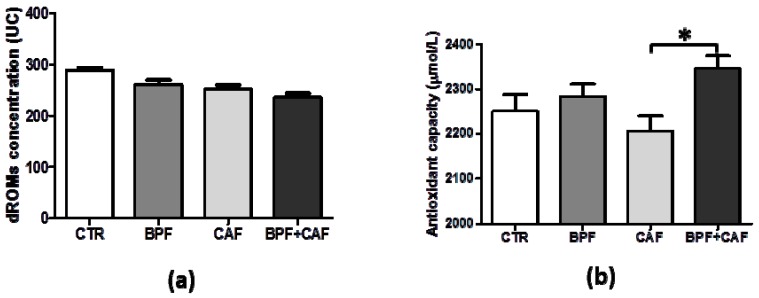
Plasmatic values of Diacron-reactive oxygen metabolite (dROM) (**a**) and biological antioxidant capacity (BAP) (**b**) test in control (CTR), bergamot polyphenol fraction (BPF), cafeteria (CAF) and BPF + CAF rats. Data are expressed as mean ± standard deviation (SD; *n* = 12). Statistical differences were evaluated by two-way ANOVA followed by Tukey’s multiple comparisons test (* *p* < 0.05).

We analyzed the trend of both oxidative stress (d-ROMs) and antioxidant capacity efficiency (BAP) in plasma of CTR, BPF, CAF and BPF + CAF rats. The analysis of the plasmatic oxidative balance shows that the oxidative index is decreased in all treated groups compared to the control (Figure 1a) while the antioxidant capacity effectiveness is considerably decreased in the CAF group and strengthened in the animals treated with BPF (Figure 1b). These results indicate that the CAF diet induces a perturbation of the oxidative equilibrium with an antioxidant capacity depletion as overused. On the contrary, BPF treatment enhances the plasmatic ability to neutralize the oxidative insults, mainly in the case of redox disturbance due to the CAF diet (Figure 1b).

### 3.3. Antioxidant Enzymes’ Expression

**Figure 2:**
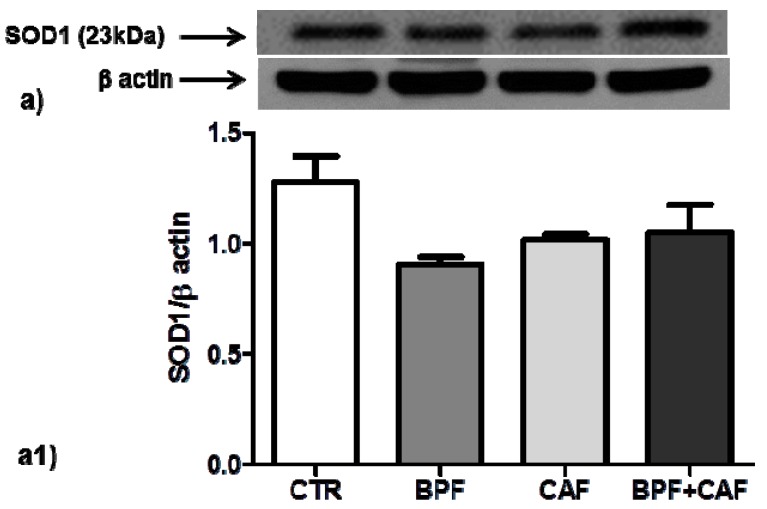
SOD1 expression in rat kidney. Western blotting of SOD1 (**a**) in the kidney extracts of CTR, BPF, CAF and BPF + CAF rats; (**a1**) shows the densitometric quantification of the blots. Protein loading was verified by using the anti-β actin antibody. Data are means ± standard deviation (SD) of five determinations for each animal (*n* = 3). Statistical differences were evaluated by two-way ANOVA followed by Tukey’s multiple comparisons test.

**Figure 3:**
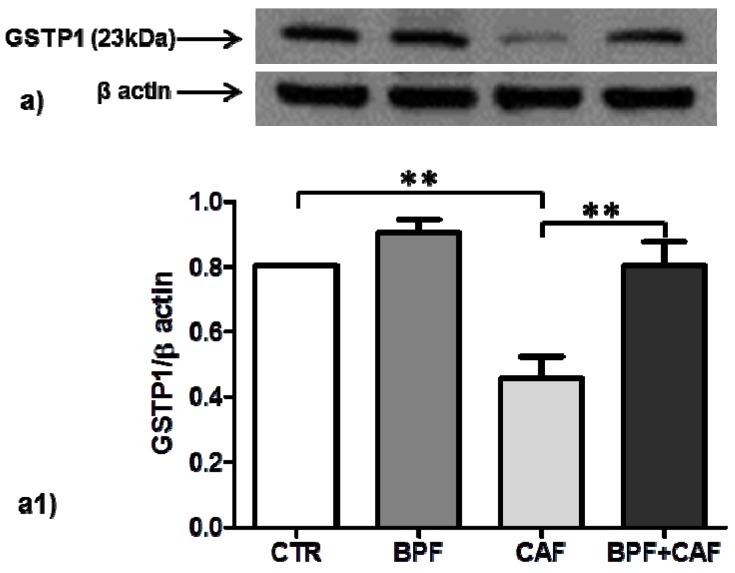
Monomeric GSTP1 expression in rat kidney. Western blotting of monomeric GSTP1 form (**a**) in the kidney extracts of CTR, BPF, CAF and BPF + CAF rats; (**a1**) shows the densitometric quantification of the blots. Protein loading was verified by using the anti-β actin antibody. Data are means ± SD of five determinations for each animal (*n* = 3). Statistical differences were evaluated by two-way ANOVA followed by Tukey’s multiple comparisons test (** *p* < 0.001).

**Figure 4:**
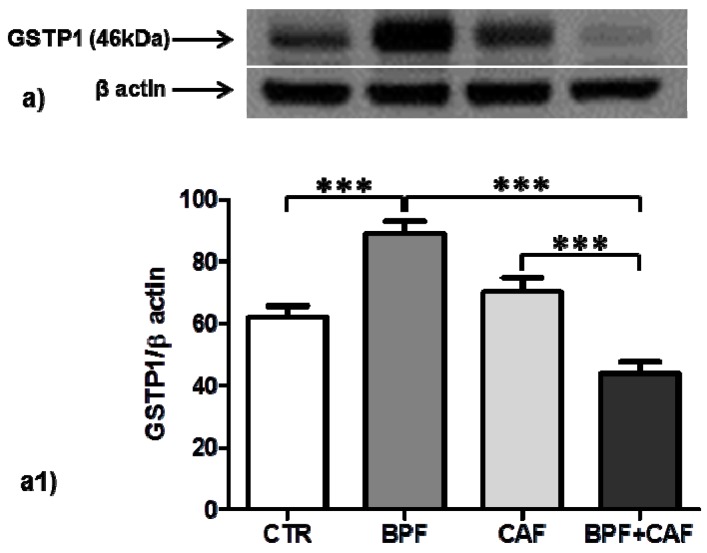
Dimeric GSTP1 expression in rat kidney. Western blotting of dimeric GSTP1 form (**a**) in the kidney extracts of CTR, BPF, CAF and BPF + CAF rats; (**a1**) shows the densitometric quantification of the blots. Protein loading was verified by using the anti-β actin antibody. Data are means ± SD of five determinations for each animal (*n* = 3). Statistical differences were evaluated by two-way ANOVA followed by Tukey’s multiple comparisons test (*** *p* < 0.0001).

We examined the expression of two important cytoplasmatic antioxidant enzymes, SOD1 and GSTP1, in CTR, BPF, CAF and BPF + CAF rats. The expression of SOD1, the most important preventive antioxidants, shows no significant changes in any of the treated groups (Figure 2). Concerning GSTP1, the expression of GSTP1 monomer (23 kDa), the form with antioxidant and proliferative activity, is increased by BPF treatment while considerably and significantly decreased by the CAF diet (Figure 3). The expression of the enzymatically active dimeric form (46 kDa) does not differ significantly in the CAF diet group while it is increased in BPF group and is decreased in BPF + CAF group (Figure 4).

### 3.4. Apoptotic Pathways

**Figure 5:**
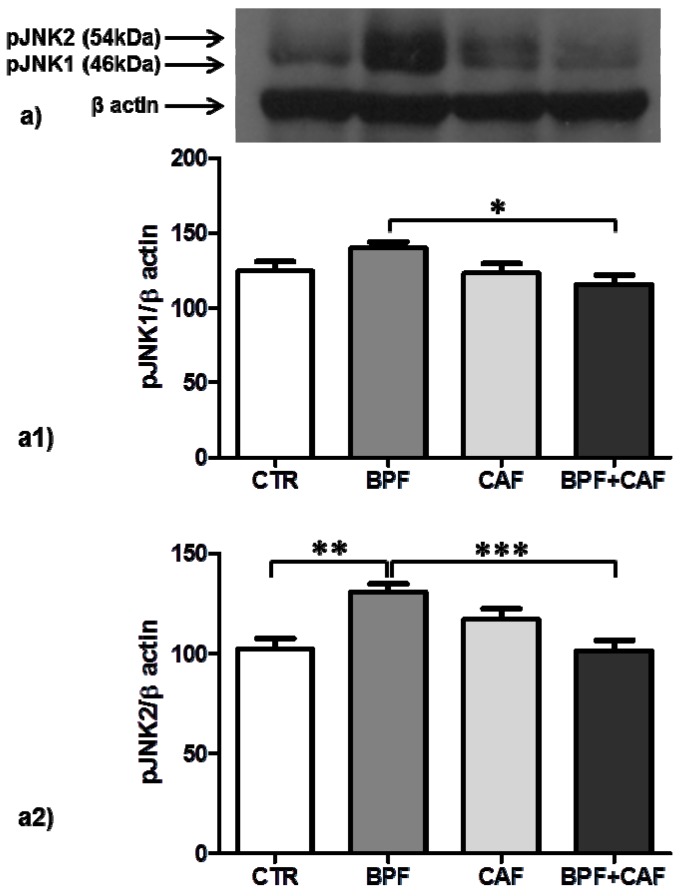
pJNK expression in rat kidney. Western blotting of pJNK (**a**) in the kidney extracts of CTR, CTR, BPF, CAF and BPF + CAF rats; (**a1**,**a2**) show the densitometric quantification of the blots. Protein loading was verified by using the anti-β actin antibody. Data are means ± SD of five determinations for each animal (*n* = 3). Statistical differences were evaluated by two-way ANOVA followed by Turkey’s multiple comparisons test (* *p* < 0.05; ** *p* < 0.001; *** *p* < 0.0001).

**Figure 6:**
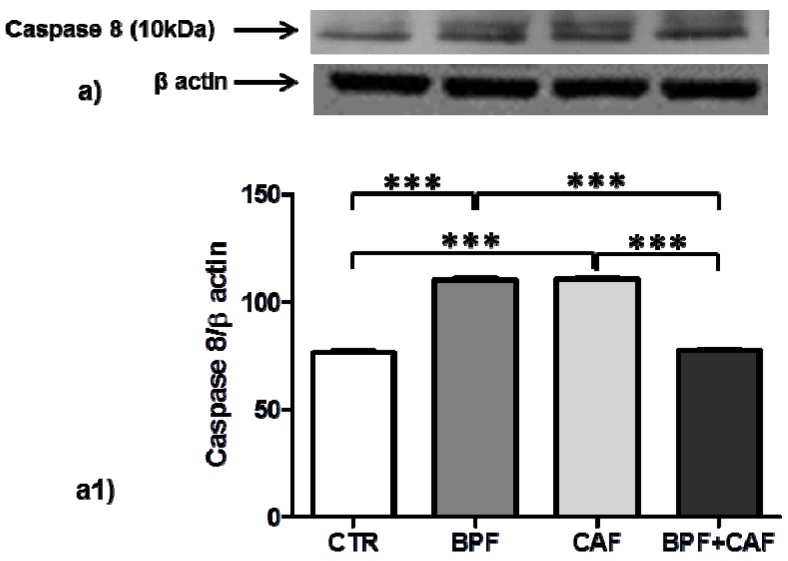
Caspase 8 expression in rat kidney. Western blotting of Caspase 8 active fragments cleaved (**a**) in the kidney extracts of CTR, BPF, CAF and BPF + CAF rats; (**a1**) shows the densitometric quantification of the blots. Protein loading was verified by using the anti-β actin antibody. Data are means ± SD of five determinations for each animal (*n* = 3). Statistical differences were evaluated by two-way ANOVA followed by Tukey’s multiple comparisons test (*** *p* < 0.0001).

**Figure 7:**
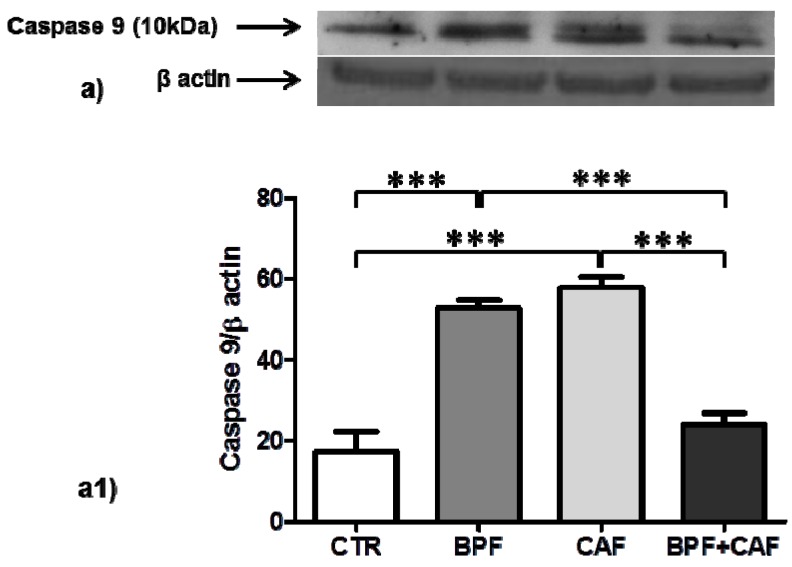
Caspase 9 expression in rat kidney. Western blotting of Caspase 9 active fragments cleaved (**a**) in the kidney extracts of CTR, BPF, CAF and BPF + CAF rats; (**a1**) shows the densitometric quantification of the blots. Protein loading was verified by using the anti-β actin antibody. Data are means ± SD of five determinations for each animal (*n* = 3). Statistical differences were evaluated by two-way ANOVA followed by Tukey’s multiple comparisons test (*** *p* < 0.0001).

In CTR, BPF, CAF and BPF + CAF rats, we analyzed apoptosis activation by evaluating the expression of pJNK (detected as double bands: pJNK1, 46 kDa; pJNK2, 54 kDa), apoptotic extrinsic pathways by evaluating the expression of caspase 8, and apoptotic intrinsic pathways by evaluating the expression of caspase 9. The expression of pJNK1, marker of apoptotic aspecific activation, is increased only in the CTR+BPF group (Figure 5a1) while the expression of pJNK2, preferentially activated by oxidative stress, is increased in all treated groups, significantly only in the BPF group (Figure 5a2). Both Caspase 8 (marker of apoptotic extrinsic pathways) and Caspase 9 (marker of apoptotic intrinsic pathways) show the same expression profile: a significant upregulation in BPF and CAF groups while no activation in the BPF + CAF group (Figure 6 and Figure 7). These results indicate that the CAF diet induces apoptotic injuries mitigated by BPF treatment.

## 4. Discussion

This study was designed to evaluate if the CAF diet, a robust model of human metabolic syndrome, can affect the plasmatic oxidative balance and can influence renal tissue alterations, in terms of both oxidative damage (altered expression antioxidant enzymes) and apoptotic pathways (intrinsic/extrinsic) activation. In parallel, the antioxidant and cytoprotective effects of the Citrus flavonoids from bergamot, BPF, were evaluated. Our results showed relevant alterations in redox status in the CAF-fed rats compared to controls and revealed that BPF treatment enhances the plasmatic and cellular ability to neutralize the oxidative insults, mainly in the case of redox disturbance due to the CAF diet. 

The CAF diet is considered to be the most appropriate regime to induce in rodents severe obesity, glucose intolerance, insulin resistance, high plasma triglyceride levels, and liver steatosis [30,32]. In addition, the CAF diet reflects the typical food of Western societies, thus representing an important model of diet-induced obesity in humans [40]. We used a CAF diet-fed rat, an excellent experimental model of obesity-induced organ damage particularly useful for analyzing the intricate link between obesity and kidney damage. Most of the rodent models of diet-induced obesity were obtained by high fat (HF) diets [41,42], but our experimental model has been shown to induce hyperphagia and obesity in rodents to a greater extent than the classic HF diets [43,44]. In our study, we have verified that the CAF rats were already obese after eight weeks and presented significant changes in blood glucose and triglyceride levels after fourteen weeks. In our experimental model, steatosis induced by CAF diet and fat content of cells have been extensively analyzed in a precedent study [30]; however, markers like adipokines (leptin, adiponectin) have not yet been evaluated. Despite the state of overt obesity and the clear metabolic alterations, after 14 weeks of CAF diet, renal function was still preserved at this time point. 

Obesity is characterized by complex metabolic abnormalities and acts as an important risk factor for renal diseases as highlighted by several studies in which BMI has been correlated with the risk of both development and progression of CKD [7,8], nephrolithiasis [45] and renal cancer [46,47,48]. Paradoxically, obesity has been consistently associated with lower mortality rates in patients with advanced CKD [49,50] and ESRD [51,52]. This apparent discrepancy makes it particularly important to study the mechanisms, still unknown, by which obesity can cause or exacerbate CKD. It is well known that the comorbidities typical of obesity, such as diabetes and hypertension, have a deleterious effect on renal function, but there are also direct effects on kidney tissue induced by the endocrine activity of the adipose tissue [6]. Indeed, some studies have highlighted a BMI-independent association between abdominal obesity and mortality in patients with ESRD [11] and kidney transplant [12], suggesting a direct role of visceral adiposity.

The link between obesity and renal damage could be represented by oxidative stress. Some authors have reported that obesity and obesity-induced insulin resistance are associated with systemic oxidative stress [53] and, in several models, the mechanism has been identified by the activation of the c-Jun N-terminal kinase pathway [54,55,56,57,58]. Our results indicate that the CAF diet induces a perturbation of the plasmatic oxidative equilibrium with the repairing intervention of the antioxidant capacity that is thus decreased as overutilized. It is well known that an oxidative insult determine a depletion of non-enzymatic antioxidants since the ROS species neutralization implies their consumption [59]. The occurrence that the efficacy of total plasma antioxidant capacity is significantly depleted in relation to metabolic disorders (such as diabetes, obesity, and dyslipidemia) had already been highlighted by our research group as the first detectable event of a redox disturbance in humans [37]. Further evidence of this mechanism is that BPF treatment enhances the plasmatic ability to neutralize the oxidative insults, mainly in the case of redox disturbance due to the CAF diet. 

Concerning enzymatic antioxidants, regulation mechanisms are more complex: in the case of low/medium oxidative stimulation, enzymatic antioxidant activity can increase, but, if oxidative stress is persisting, or its level is very high, the damage caused to proteins becomes profound and a decreased expression/activity may occur via direct oxidative damage of the molecules and/or oxidative-altered gene expression. Alterations in the enzymatic antioxidant defense mechanisms are reported in obesity models including human, but the described scenario is very intricate [60]. In obese mice, expression levels and activities of antioxidant enzymes decreased particularly at adipose tissue level [61] while the levels of antioxidant enzymes in hamsters were not greatly modified by CAF diet-induced obesity [62]. In renal tissues of our experimental model, we examined the expression of two key cytoplasmatic antioxidant enzymes, SOD1 and GSTP1. The expression of SOD1, the most important preventive antioxidants that catalyze the dismutation reaction of superoxide anion to the more stable hydrogen peroxide [63,64], shows no significant changes in relation to CAF or BPF treatments while the expression of GSTP1 monomeric form, with antioxidant and proliferative activity, is increased by BPF treatment while considerably and significantly decreased by the CAF diet. These results corroborate the recent genomic evidence that highlights as the CAF diet induced alterations in the white adipose gene transcriptome, with important suppression of glutathione-related genes and pathways involved in mitigating oxidative stress [65]. The non-catalytic role of GSTP1 monomeric form plays a key role in stress response cellular pathways acting as a JNK-proliferative pathway activator [66] in both humans and rodents [67,68].

Several studies have shown that GSTP1 acts as a stress response protein that multimerizes through disulfide cross-links, if affected by oxidative stress, and loses its ability to bind JNK, causing an increase in JNK-apoptotic pathways [67]. In renal tissue, by analyzing the apoptotic signal, we found that CAF diet induces apoptotic injuries induced by oxidative events, mitigated by BPF treatment. In particular, the expression of pJNK1, marker of apoptotic aspecific activation, shows no significant changes in CAF diet-fed rat while the expression of pJNK2, preferentially activated by oxidative stress, is increased in all treated groups, significantly only in the CTR+BPF group. JNK is a mitogen-activated protein kinase (MAPK) and plays both physiological and pathophysiological role in cells. Several lines of evidence propose that JNK is a crucial mediator in oxidative stress-induced apoptotic cell death in obesity and insulin resistance [69,70]. Our result confirmed this finding; indeed, in our obesity model, we detected a specific and robust pJNK2 activation, which supports the hypothesis that enhanced JNK activity is a critical mechanism underlying the apoptotic response to oxidant injury in obesity. Apoptosis signaling has been widely classified into extrinsic (initiated by death receptors) and intrinsic (initiated by mitochondrial events) pathways, and pJNK plays a central role in both of these pathways [71,72]. The apoptotic extrinsic pathway activation is mediated by caspase-8 [73] and the activation of the intrinsic pathway is mediated by caspase-9 [74]. In the rat model for kidney disease, the apoptotic process is associated with both intrinsic and extrinsic pathways [75]. In the present study, by analyzing both the extrinsic and intrinsic apoptotic pathways, we found that, in kidney tissue, both caspases 8 and 9 show a significant upregulation in CAF obese group and no activation in the BPF + CAF group, confirming the apoptotic response to oxidant injury in obesity and the protective effect exerted by the BPF. Our results support the existing data on the flavonoids nephroprotective effect mainly exerted on oxidative perturbations affecting apoptotic pathways [76]. 

An interesting element that emerges from our results is represented by the “negative” effect exerted by the BPF in the absence of redox stimulation. Indeed, in this case, BPF induces a pro-oxidant effect as made evident by the upregulation of antioxidant enzymes. In addition, the obese rats treated with BPF show an improvement of their apoptotic profile, while the controls treated with the same BPF doses show a worsening of their apoptotic profile. This suggests that high doses of bergamot polyphenols, well-tolerated at the liver level and sufficient to balance pro-oxidative effects of CAF diet [30], might be actually slightly nephrotoxic, if applied in association with the normocaloric diet. Accordingly, such an undesirable effect of flavonoids on renal tissues has been recently highlighted. Despite its beneficial and antioxidant activity in some types of kidney disease, high doses of green tea polyphenols exerted clearly nephrotoxic effects in streptozotocin-induced diabetic mice [77] as well as in chronic renal failure by reducing the elimination of nephrocardiovascular toxins [78]. These data underscore again that the use of antioxidant (but also other) supplements should be reserved for situations of proven lack. Indeed, in humans, nutraceutical supplementation has induced improvements in blood lipid profile but not in BMI if not associated with diet [79,80] while consumption of flavonoid-containing foods was inversely associated with both obesity and markers for metabolic syndrome [81].

## 5. Conclusions

Data collected in the present study show that the link between obesity and renal damage could be represented by oxidative stress. Moreover, we highlighted the alteration of the plasma antioxidant capacity as the first detectable and reversible event. Our results can contribute to better understand mechanisms underlying the relationship between obesity and renal tissue damage.

## 6. Clinical Perspectives

Obesity is a complex, multi-factorial pathology and represents a potent risk factor for kidney disease. The aim of our study was to assess if the CAF diet, a useful model for studying human metabolic syndrome, can induce renal tissue damage in terms of both oxidative perturbation (altered expression antioxidant enzymes) and apoptotic pathway (intrinsic/extrinsic) activation. In parallel, we evaluated the BPF cytoprotective effects. Our results showed significant alterations in redox status in the CAF-fed rats and revealed that BPF treatment enhances the plasmatic and cellular ability to neutralize the oxidative insults, mainly in the case of redox disturbance due to the CAF diet. A deeper understanding of the mechanisms underlying the relationship between obesity and progression of renal tissue damage can provide new treatment possibilities.

## Figures and Tables

**Table 1:** Body weight and biochemical profiles of Wistar rats at 22 weeks of age, fed control (CTR) or cafeteria (CAF) diet, supplement or not with bergamot polyphenol fraction (BPF; 50 mg/kg/rat) for 14 weeks. Data are expressed as Mean ± standard deviation (SD; *,^#^, *p* < 0.05 and **,^##^, *p* < 0.01 when compared with CTR animals or CAF animals, respectively).

Body Weight and Biochemical Profiles	CTR	BPF	CAF	BPF + CAF
**Body weight (g)**	466.0 ± 34.1	460.4 ± 49.6	615.0 ± 29.3 **	556.7 ± 55.7 **^#^**
**Glucose (mg/dL)**	230.0 ± 27.6	266.5 ± 28.4	350.86 ± 30.2 **	310.4 ± 47.6 **^#^**
**Triglycerides (mg/dL)**	57.0 ± 18.7	55.0 ± 23.6	86.3 ± 14.6 *	61.9 ± 14.6 **^##^**
**Cholesterol, total (mg/dL)**	92.8 ± 12.2	89.2 ± 12.0	90.5 ± 11.8	73 ± 11.7 **^#^**
**HDL cholesterol (mg/dL)**	63.6 ± 11.2	63.4 ± 12.0	61.6 ± 9.3	50.8 ± 4.9 **^#^**
**LDL cholesterol (mg/dL)**	25.2 ± 9.2	19 ± 7.9 *	18.1 ± 5.7 *	13.1 ± 4.7 **^#^**
**Creatinin (mg/dL)**	0.8 ± 0.12	0.74 ± 0.11	0.8 ± 0.08	0.8 ± 0.07
